# Digitally Delivered Interventions to Improve Nutrition Behaviors Among Resource-Poor and Ethnic Minority Groups With Type 2 Diabetes: Systematic Review

**DOI:** 10.2196/42595

**Published:** 2024-02-01

**Authors:** Nazgol Karimi, Rachelle Opie, David Crawford, Stella O’Connell, Kylie Ball

**Affiliations:** 1 Institute for Physical Activity and Nutrition, School of Exercise and Nutrition Sciences Deakin University Melbourne Australia

**Keywords:** digitally delivered, eHealth, type 2 diabetes, nutrition, socioeconomically disadvantaged, mobile phone

## Abstract

**Background:**

Resource-poor individuals, such as those with a low income, are disproportionately affected by diabetes and unhealthy eating patterns that contribute to poor disease self-management and prognosis. Digitally delivered interventions have the potential to address some of the barriers to healthy eating experienced by this group. However, little is known about their effectiveness in disadvantaged populations.

**Objective:**

This systematic review is conducted to assess the effectiveness of digitally delivered interventions in improving nutritional behaviors and nutrition‐related health outcomes among disadvantaged people with type 2 diabetes (T2D).

**Methods:**

MEDLINE complete, Global Health, Embase, CINAHL complete, Informit Health, IEEE Xplore, and Applied Science and Technology Source databases were searched for studies published between 1990 and 2022 on digitally delivered nutrition interventions for disadvantaged people with T2D. Two reviewers independently assessed the studies for eligibility and determined the study quality using the Cochrane Risk-of-Bias Assessment Tool. The Behavioral Change Technique Taxonomy V1 was used to identify behavior change techniques used in the design of interventions.

**Results:**

Of the 2434 identified records, 10 (0.4%), comprising 947 participants, met the eligibility criteria and were included in the review. A total of 2 digital platforms, web and messaging services (eg, SMS text messaging interventions or multimedia messaging service), were used to deliver interventions. Substantial improvements in dietary behaviors were reported in 5 (50%) of the 10 studies, representing improvements in healthier food choices or increases in dietary knowledge and skills or self-efficacy. Of the 10 studies, 7 (70%) examined changes in blood glucose levels, of which 4 (57%) out of 7 achieved significant decreases in hemoglobin A_1C_ levels ranging from 0.3% to 1.8%. The most frequently identified behavior change techniques across all studies were *instruction on how to perform the behavior*, *information about health consequences*, and *social support*.

**Conclusions:**

This review provided some support for the efficacy of digitally delivered interventions in improving healthy eating behaviors in disadvantaged people with T2D, an essential dietary prerequisite for changes in clinical metabolic parameters. Further research is needed into how disadvantaged people with T2D may benefit more from digital approaches and to identify the specific features of effective digital interventions for supporting healthy behaviors among disadvantaged populations.

**Trial Registration:**

PROSPERO International Prospective Register of Systematic Reviews CRD42020149844; https://www.crd.york.ac.uk/prospero/display_record.php?RecordID=149844

## Introduction

### Background

Type 2 diabetes (T2D) is a common and increasingly prevalent noncommunicable disease [1]. This disease, considered an epidemic of the 21st century, is a serious challenge for public health, affecting the health of millions of people worldwide [2]. The burden of T2D is growing, specifically among resource-poor and ethnic minority groups [2,3]. These groups are more likely to experience higher rates of suboptimal glycemic control, diabetes-related complications, hospitalizations, as well as increased mortality and morbidity compared with more advantaged populations [4,5]. T2D and its associated complications pose a huge economic cost to the health system and patients through rising health care costs associated with direct medical costs and loss of work and income [5]. The direct and indirect annual costs of diabetes (with approximately 90% comprising T2D) and its various complications account for >2 million deaths each year and cost >US $827 billion worldwide [6,7].

### Diabetes Self-Management

Self-management is considered to be the hallmark of diabetes control [8]. T2D self-management involves a combination of nutritional, lifestyle, and medication therapies to optimize glycemic control and reduce complications associated with diabetes. A total of seven self-care behaviors are important in T2D self-management: (1) healthy eating, (2) being physically active, (3) taking medication, (4) monitoring blood glucose, (5) reducing risk, (6) healthy coping, and (7) problem-solving [9].

### Healthy Eating

Healthy eating is a key component of T2D self-management [10]. Evidence shows that improved diet quality in people with T2D can decrease hemoglobin A_1c_ (HbA_1c_) levels to a similar or even better level than the provision of medication [11]. Healthy eating remains an important element of the overall treatment plan for people with T2D, although medication is necessary. In addition, improved diet quality may prevent obesity, high blood pressure, and abnormal lipid profile, which in turn decrease the risk of cardiovascular diseases in people with T2D [11].

People who are socioeconomically disadvantaged, including those with lower incomes, lower education levels, or lower status occupations, are at an increased risk of unhealthy dietary intake, with diets characterized by fewer fruits and vegetables and higher intakes of less healthy discretionary foods and beverages [12-14]. The less optimal eating behaviors observed among socioeconomically disadvantaged groups have been attributed to a range of factors, including lower levels of nutrition knowledge, lower prioritization of health during food selection, less access to nutrition education, lower social support, limited access to higher quality foods, and cost-related barriers [3,15-19]. Furthermore, these barriers to healthy eating partly explain the poor clinical outcomes and higher incidence of diabetic complications in these groups [20].

### Limitations of In-Person Diabetes Self-Management Education

Generally, supporting people in T2D management, including healthy eating, involves one-on-one or group-based, in-person education provided by a health care provider (HCP). These clinical approaches can provide the education and support needed by people with T2D. However, the proportion of patients receiving any type of diabetes education or behavioral support is low globally. For instance, only 11% of patients with T2D in the United Kingdom, 23% to 66% in the United States [21], and 40% in Australia receive diabetes education, and this proportion is even lower for disadvantaged populations [22]. This has been attributed to factors such as the limited availability and accessibility of HCPs, lack of time, travel costs, limited insurance reimbursement for diabetes self-management (DSM) education, or disabilities that make travel to DSM education services challenging [3].

### Potential for New Digital Technologies

Digital technologies provide a unique delivery mode for promoting T2D self-management. These include mobile technologies, such as tablets, phones, smartphones, and physical activity tracking devices, to help and improve public health practices [23]. These technologies can provide remote access to information that forms the basis of the current in-person T2D self-management support programs.

Digitally delivered health interventions include any web-based application that uses processing or communication via digital technologies to facilitate ≥1 aspect of self-management. These include technologies that can promote (1) self-tailoring goals without requiring continuous professional input, (2) decision-making, (3) emotional management, (4) resource use, (5) action planning for behavior change, or (6) problem-solving [22]. These interventions provide opportunities to overcome some of the barriers of in-person T2D self-management education such as limited access to HCPs and could be a more effective approach to helping people learn self-management skills [24]. Moreover, digital interventions are easily distributable and not limited to a specific location; therefore, they can be delivered in clinics and community health centers, at home, or on the move at a time of greatest convenience to the users [22,24,25]. In addition, they can potentially offer ongoing support and provide extensive intervention as needed [24]. Furthermore, these interventions can accommodate different literacy levels, learning styles, and user-specific preferences and needs (eg, linguistic or cultural background) [26].

### Importance of This Review

Many earlier systematic reviews investigating digitally delivered interventions for T2D self-management reported positive outcomes [22,24-37]. However, in nearly all of these studies, remote technologies were used to aid data collection (eg, records of medication, physical activity, or diet) and as a supplement to other intervention components rather than the core intervention component. Furthermore, to the best of our knowledge, all existing reviews, except the one by Dening et al [31] that specifically focused on the effect of digitally delivered interventions on nutrition behaviors such as the intake of vegetables and fruits, predominantly combined different target behaviors (eg, medication adherence, diet, and physical activity) [24,25,38,39] or examined single nutrition‐related health outcome (eg, weight loss) [40] in general (not specifically disadvantaged) populations.

We were able to identify 2 reviews that focused on evaluating the effects of digitally delivered interventions for disadvantaged people with T2D or a mixed sample of both type 1 and type 2 diabetes [25,26], although the main focus of these reviews was on HbA_1c_ changes, and none of these specifically addressed nutritional outcomes.

Although healthy nutritional behavior is an important part of DSM and nutrition education and support is an urgent need for people with T2D, digitally delivered interventions to date have chiefly focused on overall self-management; although some studies have included a dietary component within the intervention package, the assessment of dietary behavior changes or adherence remains scarce. To date, we are aware of only 1 review by Dening et al [31] that has investigated the effects of digital interventions on changes in dietary behavior in people with T2D, and we could not find any analogous review that targeted the assessment of dietary behavior changes in disadvantaged populations. Conducting systematic reviews with a focus on people of lower socioeconomic position is important because the health intervention features that work well in well-resourced settings may not be available or effective for disadvantaged groups [25,26]. Therefore, a considerable evidence gap remains in investigating the effects of digitally delivered interventions on nutrition behaviors and nutrition-related health outcomes among socioeconomically disadvantaged people with T2D, and this systematic review was conducted to address this gap.

To be effective, T2D self-management interventions need to help people increase their understanding of their condition and alter their adherence patterns to effective self-management behaviors (eg, healthy eating). To achieve this aim, many interventions apply behavioral theories and behavior change techniques (BCTs). BCTs are “observable, replicable, and irreducible” components of interventions [41,42]. Extracting information about the theoretical framework and behavioral content of interventions using an established behavior change taxonomy can provide insight into the active ingredients of digital interventions and can help guide future intervention development. Therefore, as part of the aim of this review, we extended the scope of previous systematic reviews by identifying the behavioral theory and techniques underpinning each intervention.

## Methods

### Reporting Guidelines

This systematic review was reported following the PRISMA (Preferred Reporting Items for Systematic Reviews and Meta-Analyses) statement [43]. The review protocol was registered at PROSPERO (2019#CRD42020149844). Study searches, selection, and synthesis were guided by the population, intervention, control, and outcomes (PICO) statement described in the *Eligibility Criteria* Section.

### Eligibility Criteria

The eligibility criteria for the systematic review and search strategy were defined a priori to minimize bias in the selection of studies. The criteria encompassed various aspects, including population characteristics, intervention types, control parameters, study outcomes, acceptable study types, and intervention duration. Below is a detailed breakdown of each criterion:

- Population: studies were eligible for inclusion if they were original research studies on adult participants aged ≥18 years who were clinically diagnosed with T2D and were from disadvantaged populations (≥50% of the sample). Disadvantaged populations included people of low socioeconomic status (SES; ie, people with low educational level, low income, low occupational status, or people living in low SES residential areas) or members of a racial or ethnic minority group. The latter were included because these groups often experience socioeconomic disadvantage [44], are at increased risk of chronic disease [45], and face significant challenges in accessing health promotion services [46-49]. Studies were deemed ineligible if the participants were children aged <18 years, women with gestational diabetes, or people with prediabetes. No criteria were established for the maximum and minimum number of participants in each study.
- Intervention: all types of mobile- or web-based interventions that delivered nutritional knowledge or skills were considered eligible. We defined interventions as mobile based or web based if participants directly received information and skills from digital devices and interfaced with the World Wide Web. On the basis of this definition, studies that used digital devices only as a data collection tool and did not provide any information or feedback on participants’ inputs through the web or telehealth interventions that only provided access to a health professional without further digital support were not deemed mobile based or web based and were, therefore, ineligible for the review.
  Studies with combined or composite interventions, such as those that provided nutritional information as a component of a multifaceted mobile- or web-based intervention, were considered eligible.
- Control: no limitations were imposed on the control group, and studies with or without a control group were considered eligible.
- Outcomes: intended study outcomes for eligibility were measures focused on nutrition-related behaviors (eg, food intake, nutritional knowledge, and skills) or nutrition-related health outcomes (eg, glycemic control as indicated by HbA_1c_ levels and biomarkers such as weight and BMI). Studies with outcomes related to changes in blood lipid levels, exercise levels, and medication adherence were not included in this review.
- Study type: randomized and nonrandomized controlled trials were considered eligible for inclusion. However, protocol papers, letters, and conference proceedings were excluded. The reference lists of systematic reviews were searched for relevant papers.
- Intervention duration: no limitation was placed on the duration of the intervention or follow-up period, and studies of any duration were considered eligible for inclusion.

### Information Sources

Relevant papers were obtained by systematic search using the following electronic databases: MEDLINE complete, Global Health, Embase, CINAHL complete, Informit Health, IEEE Xplore, and Applied Science and Technology Source. Additional publications were identified from the reference lists of the original papers. In some instances, when we found a relevant conference abstract or protocol paper but not the entire result paper, we contacted the study authors to identify additional studies. Each source was last searched on April 6, 2022.

### Search Strategy

The search strategy was developed in cooperation with Deakin University’s expert librarian team. The strategy comprised 4 main category blocks with medical subject headings terms related to digital health, healthy eating, SES, and diabetes. The search was limited to peer-reviewed studies that were written in the English language only and published between January 1990 and April 2022. Full search strategies for the MEDLINE complete, Global Health, CINAHL complete, and Applied Science and Technology Source databases can be found in Multimedia Appendix 1.

### Selection Process

The reference management software EndNote X8 (Clarivate) [50] was used to merge the search results and remove the duplicate papers. All papers were then exported to a web-based systematic review management tool, Covidence (Veritas Health innovation) [51], for other stages of screening, data extraction, and quality assessment. Using the inclusion and exclusion criteria, all potentially relevant papers were independently screened by 2 reviewers (NK and DC or KB) for eligibility based on titles and abstracts. If deemed potentially eligible, the full-text publication was extracted and reviewed by 2 reviewers (NK and DC or KB) to be included in the systematic review. The other team members were consulted in cases of uncertainty.

Finally, studies that fulfilled the eligibility criteria for the review were examined by extracting data on the key criteria in a tabulated form for comparison, qualitative evaluation, and discussion.

### Data Extraction and Synthesis of Results

Data extraction was performed by 2 independent authors (NK and DM or KB). Data were extracted from the final selected studies using the following parameters: basic characteristics (including author, date, country, study design, sample size, gender, age, race and ethnicity, attrition, study location, and delivery modes), intervention characteristics (including the type of digital intervention and intervention components, behavior change theory, BCTs, control group protocol, total study duration, and follow-up), and research outcomes (including dietary intake; nutritional knowledge or skills; diet-related health outcomes, such as glycemic control as indicated by HbA_1c_ levels; and biomarkers, such as weight and BMI). Owing to insufficient data and heterogeneity of the included studies, conducting a formal meta-analysis was not possible. Therefore, the findings of this systematic literature review are presented as a narrative summary.

### Reporting Bias Assessment

The methodological quality of each paper was independently assessed by 2 reviewers (NK, KB, or RO). Disagreements were resolved by consensus, consultation with a third reviewer (KB, or DC), or discussion in a group meeting of coauthors where the final decisions were made. The Cochrane Collaboration tool [44] was used to assess the risk of bias in all included studies [52].

## Results

### Study Selection

**Figure 1:**
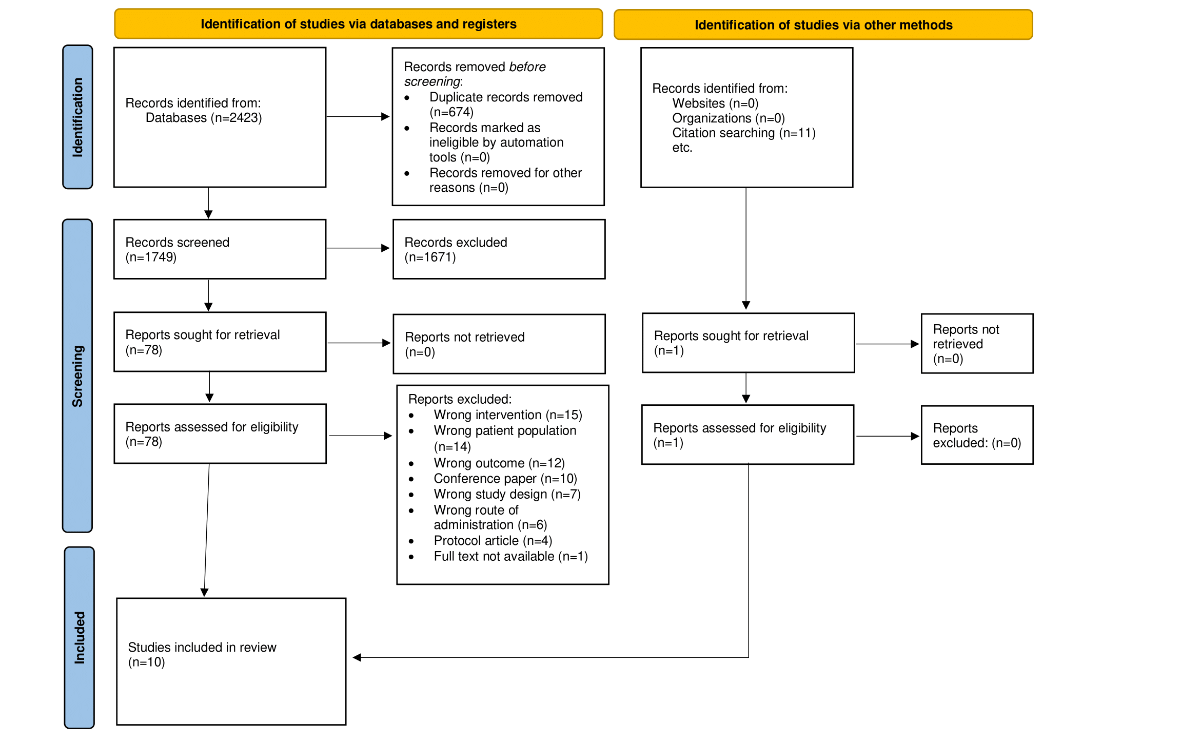
Flowchart of the study selection process.

The initial search resulted in 2434 studies, of which 674 (27.69%) duplicates were removed and 1760 (72.31%) studies were screened against the title and abstract. Furthermore, of the 1760 studies, 1681 (95.51%) were excluded after preliminary screening for not meeting the inclusion criteria. Of the remaining 79 studies reviewed completely, 10 (13%) met the inclusion criteria and were included in this review. Figure 1 displays the results of study selection and the number of included and excluded studies.

Most studies excluded from the review were telehealth interventions. The reason for their exclusion was that the main aim of these studies was to enable health professional–patient interaction at a distance rather than providing patients with knowledge or skills to improve healthy eating behaviors as part of T2D self-management education. Interventions that used mobile apps only for daily recording of diet were also excluded because they did not meet our inclusion criteria that participants received educational information and behavioral change support through the World Wide Web.

Studies with general populations only (eg, participants with average or higher SES) or with less than half of the participants belonging to low SES groups were the other common group of studies that were excluded. Other reasons for exclusion were studies with no clear dietary intervention components, studies with no diet-related outcomes, or studies focused on children or participants with type 1 diabetes only.

### Participants and Study Characteristics

Table 1 presents the characteristics of all the included studies. Out of 10 studies, most (n=7, 70%) were conducted in the United States [53-57], with 1 (10%) study conducted in the Kingdom of Saudi Arabia [58], 1 (10%) in Iran [59], and 1 (10%) in Mexico [60]. The participants of all studies were mainly recruited from the community or primary health care centers, apart from 20% (2/10) of the studies that recruited participants from a hospital emergency department [53,59]. The sample sizes ranged between 13 [55] and 506 participants [61]. The mean age of participants ranged from 45.4 years [53] to 55.8 years [57]. Regarding the length of interventions, 10% (1/10) of the studies had 12 months intervention period and 3-month follow-up period [61], 40% (4/10) of the studies had 6 months intervention period [56,57,60,62], and the remaining studies (5/10, 50%) had intervention periods lasting <3 months or for a single session [53-55,58,59] without any follow-up.

Text messaging, in the form of SMS or multimedia messaging service (MMS), and websites were the 2 digital platforms used to deliver the interventions. In 5 (50%) of the 10 included studies, the intervention was administered via text messaging only [53,54,59,61,62]. In these interventions, educational materials and regular reminders to eat healthier food options and to perform exercises were sent to participants via SMS or MMS. The option for participants to send text messages to providers or educators was also a feature of these studies. In 4 (40%) of the 10 included studies [55-58], website platforms were used to deliver T2D self-management materials. Access to tailored educational materials, videos and links to other resources, and recommended websites were some of the features of these studies. For example, 1 of these studies designed an interactive website [56], in which participants could record the type and amount of food they consumed, and the system provided them with feedback on the calories and grams of proteins, fats, and carbohydrates in their food [56]. Another study incorporated a mix of 7 weekly in-person sessions and 6 months of delivering SMS text messages and MMS messages to reinforce content and promote participants’ intention and maintenance of behavior change [60].

Participants’ access to computers, mobile phones, and the internet was a barrier addressed in some of the studies. For example, in the study by Robertson et al [56], participants in the intervention group received a computer and 6 months of internet connection. Similarly, in the study by Ruggeiro et al [57], all participants in the intervention group were provided with the same type of laptop to minimize barriers to access. Furthermore, support for internet access was provided where necessary. By contrast, in 4 (40%) of the 10 studies [53,54,59,60], participants were required to own a mobile phone for inclusion in the intervention.

**Table 1:** Characteristics of the included studies.

Study and country	Study design and intervention duration	Inclusion criteria	Sample characteristics, race or ethnicity, and attrition rate	Technology type	Intervention characteristics	Intervention tailoring (eg, tailored digital design, tailored content, or personalized content)	Control group
Aligholipour et al [59], 2019; Iran	2-arm randomized controlled trial for a period of 3 months	Having T1D^a^ No severe heart disease No renal disease No admission because of ketoacidosis and nonketogenic hyperosmolar syndrome No severe visual, hearing, and cognitive defect Resident of Tabriz or near villages Having a cell phone with the Telegram app	N=66 (33.3% female), intervention group (n=33) and control group (n=33), and mean age 44.91 (SD 11.8) years T2D^b^%: ND^c^ Study setting or recruitment site: emergency department or the outpatient clinic of the hospital Race and ethnicity: 100% Iranian Attrition rate: 4.54%	Mobile phone: MMS^d^	Intervention delivery: MMS+nurse diabetes educator (as a support) Program name: ND Intervention: at least 2 daily MMS in the form of text, image, video, or GIF sent through a specific Telegram channel, to educate regarding nutrition, exercise, insulin therapy, blood sugar monitoring, foot care, and prevention of diabetes complications	Tailored design (program designed for adults with low literacy) Personalized content (tailored feedback and training messages were provided by a nurse diabetes educator)	Usual care (3 in-person educational session by a nurse diabetes educator)
Arora et al [53], 2012; United States	1-arm nonrandomized pilot trial for a period of 3 weeks	Age ≥18 years T1D or T2D Having an SMS text message–capable mobile phone and knowing how to receive SMS text messages Ability to speak and read English or Spanish	N=23 (39% female), intervention group (n=23), control group (n=0), and mean age 45.4 (SD 8.3) years T2D%: 70% Study setting and recruitment site: emergency department of the hospital Race and ethnicity: 70% Latinx or Hispanic Attrition rate: 0%	Mobile phone: SMS text messages	Intervention delivery: fully automated Program name: TExT-MED Intervention: a total of 3 unidirectional daily SMS text messages to deliver educational and motivational material, medication reminders, and healthy living challenges Trivia questions with answers were sent 1 hour after the SMS text messages and links were provided to access free diabetes management tools.	Tailored digital design (English or Spanish)	N/A^e^
Burner et al [54], 2017; United States	2-arm randomized controlled trial for a period of 3 months	HbA_1c_^f^ level ≥8 Having a text-capable mobile phone Ability to send and receive SMS texts messages	N=44 (50% female), Intervention group (n=22) and control group (n=22), and mean age ND T2D%: ND Study setting or recruitment site: medical Center Race and ethnicity: 77% Latinx Attrition rate: 18%	Mobile phone:text messages	Intervention delivery: fully automated Program name: TExT-MED+FANS Intervention: the program consisted of 2 curricula, 1 for patients and another for supporters (eg, family members or friends), and sent 2 unidirectional daily SMS text messages delivering education and motivation (1 per week) medication reminders (3 per week), healthy living challenges (2 per week), and trivia questions (2 per week) The pair of messages was sent to the patient and the supporter synchronously.	Tailored digital design (English or Spanish)	A total of 2 SMS text messages daily for patients plus a pamphlet for supporter (no SMS text message for supporters)
Fortmann et al [62], 2017; United States; the study provided phone to participants for the intervention	2-arm randomized controlled trial for a period of 6 months	Age 18-75 years T2D HbA_1c_ ≥7.5% Spanish-speaking and English-speaking Hispanic Uninsured or underinsured	N=126 (74.6% female), Intervention group (n=63) and control group (n=63), and mean age 48.4 (SD 9.0) years T2D%: 100% Study setting or recruitment site: a network of federally qualified health centers Race and ethnicity: 100% Hispanic Attrition rate: 10.3%	Mobile phone: SMS text messages	Intervention delivery: fully automated Program name: Dulce digital Intervention: a total of 2-3 SMS text messages per day (covering topics such as diabetes and its complications; the role of diet, exercise, and medication; and the importance of self-monitoring) were sent at study start, with the frequency tapering over 6 months.	Tailored content (culturally sensitive content and recipes for Hispanic population)	Usual care including visit with primary care physician, certified diabetes educator, and group DSM^g^
Moussa et al [58], 2013; Kingdom of Saudi Arabia	2-arm randomized controlled trial for a period of 1 month	Age 40-65 years T1D or T2D Low diabetes literacy	N=46 (60.9% female), Intervention group (n=23) and control group (n=23), and mean age 52 (SD 7.99) years) T2D%: 78% Study setting or recruitment site: free health clinic and community health center Race and ethnicity: 100% African American Attrition rate: 0%	Website	Intervention delivery: trained graduate research assistants+website Program name: eCare We Care Intervention: the program provided 4-week web-based diabetes education sessions delivering introduction to diabetes, eye complications, foot care, and meal planning plus providing links to freely accessible and credible government health information websites.	Tailored digital design (designed for adults with low health and electronic literacy with simple language, option for audio, clearly labeled buttons, and text and arrows for easy navigation)	Paper-based and text-only tutorial
Nelson et al [61], 2021; United States	3-arm randomized controlled trial with a 12-month intervention perio+3 months postintervention follow-up	Adults T2D Use daily diabetes medication	N=506 (54.2% female), intervention group (n=139) and control group (n=135), and mean age of intervention group 55.8 (SD 9.8) years T2D%: 100% Study setting or recruitment site: community health center locations and primary care locations Race and ethnicity: non–Hispanic White 48%, non–Hispanic Black 39.3%, non–Hispanic other races 6.3%, and Hispanic 6.3% Attrition rate: 4.8%	Mobile phone: SMS text messages	Intervention delivery: fully automated Program name: REACH Intervention: the program included daily interactive SMS text messages and tailored SMS text messages addressing medication adherence and nontailored SMS text messages supporting other self-care behaviors (ie, diet, exercise, and self-monitoring of blood glucose) for the first 6 months. Then, 3-4 self-care promotion 1-way SMS text messages were sent each week, and 1 weekly interactive SMS text message was sent until the intervention period ended (if a participant chose the low-dose option). Half of the participants assigned to receive REACH also received FAMS for the first 6 months. FAMS included monthly phone coaching to set diabetes self-care goals and improve family and friend involvement in self-care and the option to invite an adult friend or family member to receive SMS text messages about self-care goals.	Personalized content (tailored to participants’ self-identified barriers to medication adherence and their prescribed diabetes medications)	Nonintervention
Porter et al [55], 2009; United States	1-arm nonrandomized trial for 1 session	T2D First language Spanish	Intervention group (N=13) T2D%: 100% Race and ethnicity: 100% Hispanic Attrition rate: 0%	Website	Intervention delivery: fully automated Program name: Your Guide to Diet and Diabetes Intervention: the program delivered information about general overview of diabetes and food, eating for target blood glucose levels, and eating for cardiovascular health plus provided links to other resources and recommended websites.	Tailored content (culturally sensitive content and recipes for Hispanic population) Tailored digital design (Spanish)	N/A
Robertson et al [56], 2007; United States	2-arm randomized controlled trial for a period of 6 months	Age ≥18 years No renal failure English literate Able to provide informed consent	N=35 (51.4% female), intervention group (n=29) and control group (n=19), and mean age 52.5 (SD 8.6) years T2D%: 100% Study setting or recruitment site: Northern Plains Indian reservation Race and ethnicity: 100% American Indian Attrition rate: 12.12%	Website	Intervention delivery: interactive website Program name: Keya Tracker Intervention: the program provided materials about physical activity, nutrition, cultural activity, and social activity. Participants recorded their exercise and food consumption daily (for minimum 3 times per week) and recorded their cultural and social data once a week. Keya Tracker provided feedback on the calories and grams of proteins, fats, and carbohydrates of participants’ food choices.	Tailored content (culturally sensitive content with input from Northern Plains’ Indian tribal members about a list of the specific physical, cultural, and social activities)	Nonintervention
Ruggiero et al [57], 2014; United States	1-arm nonrandomized trial for a period of 6 months	T2D African American Age ≥18 years Fluent in English Receiving medication therapy for diabetes (insulin, oral agents, or both) Not pregnant or planning a pregnancy during the study period Able to provide informed consent	Intervention group (N=41, 71% female) and mean age 55.2 (SD 9.6) years T2D%: 100% Race and ethnicity:100% African American Attrition rate: 33.3%	Computer or website	Intervention delivery: health professionals+website Program name: Diabetes Island Intervention: the program provided a virtual environment for DSM education and support and a study website for communication and tracking Health professionals (as avatars) provided real-time formal and informal educational sessions. The program had a series of 10 formal presentation sessions, including 6 healthy eating topics led by a registered dietitian, 1 on medication adherence led by a pharmacist, 1 on physical activity led by an exercise researcher, and 2 on the “ABCs” of diabetes and proper foot care led by a nurse certified diabetes educator. Several dietitian-facilitated real-time discussions on healthier eating were offered in the grocery store, fast food restaurant, and home.	Tailored content (culturally sensitive content [57,60] with input from the multidisciplinary research team and feedback from the advisory committees regarding special needs of this population)	N/A
Whittemore et al [60,63], 2020; Mexico	2-arm randomized waitlist-controlled pilot trial with 7 weekly sessions and 6 months of mHealth^g^ intervention	T2D Age 21-70 years Medically stable and able to exercise HbA_1c_ >7.5% Receiving health care at a Seguro Popular clinic Access to a mobile phone	N=47 (68% female), intervention group (n=26) and control group (n=21), and mean age 55.5 (SD 9.2) years T2D%: 100% Race and ethnicity: 100% Mexican Attrition rate: 6.4%	Mobile phone: SMS text messages or MMS	Intervention delivery: registered nurse and social worker+SMS text messages Program name: ¡Sí, Yo Puedo Vivir Sano con Diabetes! Intervention: 7 weekly sessions to progressively increase the knowledge and abilities of participants to improve their metabolic control through healthier eating, and 6 months of daily SMS text or picture messages to reinforce class content and promote participants’ intention and maintenance of behavior change	Tailored content (culturally relevant content that addressed cultural misconception) Tailored digital design (designed for adults with low health literacy, SMS text messages designed for third to fourth grade reading level, - used images for most the SMS text messages, and colloquial language)	Usual care

^a^T1D: type 1 diabetes.

^b^T2D: type 2 diabetes.

^c^ND: none described.

^d^MMS: multimedia messaging service.

^e^N/A: not applicable.

^f^HbA_1c_: hemoglobin A_1c_.

^g^mHealth: mobile health.

Most studies (8/10, 80%) focused on samples belonging to an ethnic minority population [53-59,61,62], and in others (2/10, 20%), the samples were disadvantaged in terms of income level, working status, or food insecurity [59,60]. Three different approaches were used to tailor the interventions to the participants: (1) tailoring the digital design using digital elements or language suitable for people with low literacy or electronic literacy or incorporating highly visual messages, (2) adapting content to the culture of target groups, and (3) providing personalized educational content or feedback. Regarding tailoring to language, 2 (20%) of the 10 included studies offered bilingual intervention components to the participants [53,54], and in 1 (10%) study, educational materials were completely translated from English into Spanish, the first language of the participants [55]. In 5 (50%) of the 10 studies, the educational components were informed through input from the participants, multidisciplinary research teams, and feedback from advisory committees regarding patients’ special needs and cultural preferences [55-57,60,62]. Of the 10 studies, 2 (20%) provided personalized content or feedback based on participants’ special needs [59,61].

### Nutritional Content of Interventions

Of the 10 included studies, 5 (50%) [54,55,61,62,64] were fully automated interventions without HCPs’ coaching or support, and they mainly provided general healthy eating advice based on standard diabetes dietary guidelines. In these studies, recommendations and educational materials emphasized setting goals to follow a healthy diet with more vegetables and fruits, reading food labels, choosing foods from the 5 food groups, drinking water, avoiding saturated fats, and planning meals. Because most of these studies were conducted in the United States, the dietary guidelines mentioned more often across these studies were those of the American Diabetes Association and the National Diabetes Education Program. In addition to these guidelines, Porter et al [55] referred participants to the Joslin Diabetes Center website, the National Diabetes Information Clearinghouse website, the National Institutes of Diabetes & Digestive & Kidney Diseases website, the Endocrine Society website, and the Centers for Disease Control and Prevention Diabetes Public Health Resource website. Furthermore, the remaining studies [56-60], besides digitally delivering general health advice on eating well from credible sources, provided some instruction from HCPs (eg, registered nurses, diabetes educators, or nutritionists) for participants via phone or in-person visits.

### Nutritional Outcomes and Measures

Studies have explored various outcomes such as nutrition-related cognitive or behavioral outcomes (including dietary consumption, dietary self-efficacy, nutrition knowledge, and skills), nutrition-related health outcomes (including HbA_1c_ levels, fasting blood sugar [FBS], BMI, weight, and blood lipids), medication adherence, and physical activity levels. However, because this systematic review focuses only on nutrition-specific outcomes, we only selected and summarized outcomes related to nutritional behavior and nutrition-related health outcomes.

The scales used for evaluating these 2 groups of outcomes varied across studies. Arora et al [64] used the Diabetes Knowledge Test developed by the Michigan Diabetes Research Training Center and the Diabetes Empowerment Scale-Short Form. Similarly, Burner et al [54] used the Diabetes Empowerment Scale-Short Form to evaluate changes in self-efficacy and the Problem Areas in Diabetes Scale and the Summary of Diabetes Self-Care Activities (SDSCA) Scale to evaluate the diabetes-related quality of life and healthy behaviors, respectively. The SDSCA scale was also used in the study by Whittemore et al [63], and its revised version was used in the study by Ruggeiro et al [57]. The revised version of the SDSCA scale included all the original items, and additional items to assess specific nutritional content areas emphasized in the program (ie, reading food labels, choosing foods from the 5 food groups, and drinking water). Furthermore, Ruggeiro et al [57] used the Fat-Related Diet Habits Questionnaire to assess dietary intake. Moussa et al [58] and Porter et al [58] used surveys developed by their research teams specific to assessing their intervention content. Robertson et al [56] used the United States Department of Agriculture Nutritional Nutrient Database for Standard Reference to enter participants’ food records on the website. Participants entered daily food records, specifying the type and amount of food items consumed. The first and last 3 days of each participant’s nutrition entries were analyzed using the Food Processor to evaluate changes in calories and amounts of carbohydrates, protein, and fat intake. Nelson et al [61] used the Personal Diabetes Questionnaire subscale to assess the use of dietary information for decision-making, and Aligholipour et al [59] used the Persian version of Toobert’s Self-Care Activities Questionnaire.

### Intervention Effects on Nutrition-Related Cognitive or Behavioral Outcomes and Nutrition-Related Health Outcomes

Of the 10 included studies, 9 (90%) reported outcomes in the category of nutrition-related cognitive or behavioral outcomes (Table 2) [54-59,61,63,64]. Of these 9 studies, 2 (22%) reported changes in dietary consumption [53,56], with one 1-arm nonrandomized pilot trial by Arora et al [53] showing positive changes in the intervention participants. In their study, Arora et al [53] reported a 26.5% increase in the number of people reporting eating fruits and vegetables after receiving daily educational, motivational, and reminder SMS text messages.

Of 10 studies, 3 (30%), including 2 one-arm trials [53,55] and 1 randomized controlled trial [58], reported changes in nutritional knowledge and skills, and 2 of these by Porter et al [55] and Moussa et al [58] reported positive outcomes. In this group, Moussa et al [58] conducted a controlled trial on a 4-week web-based educational intervention called *eCare We Care* and reported significant (*P*=.01) improvements in intervention participants’ diabetes knowledge scores (including nutritional knowledge questions) in comparison with the control group participants who received paper-based, text-only tutorials. Porter et al [55] also reported that the *Your Guide to Diet and Diabetes* web-based intervention led to a 39% increase in diabetes-related knowledge (including nutritional knowledge questions), a 39% increase in carbohydrate counting skills, and a 31% increase in the ability to plan a meal plate.

A total of 4 studies [54,57,59,60] in the category of nutrition-related cognitive or behavioral outcomes, including 3 randomized controlled trials [54,59,60] and 1 one-arm trial [57], evaluated the effect of digitally delivered interventions on improving dietary self-efficacy, with 3 of these studies [57,59,60] showing positive impacts on at least 1 measure. Ruggeiro et al [57], for example, reported significant improvements in 1 specific subscale of the dietary self-efficacy scale (modifying meat) among participants who were provided with a virtual environment for DSM education and support and a study website for communication and tracking (*Diabetes Island*) in a 1-arm trial. However, this study did not show any significant improvement in other items of the dietary self-efficacy scale including general diet and specific diet. By contrast, Whittemore et al (using the same scale) [63] and Aligholipour et al [59] reported significant improvement in total scores of participants’ dietary self-efficacy. Nelson et al [61] evaluated the effect of daily SMS text messages supporting self-care behaviors (ie, diet, exercise, and self-monitoring of blood glucose) on the use of dietary information for decision-making using the Personal Diabetes Questionnaire scale and reported no significant changes in participants’ total scores.

The effects of interventions on improving nutrition-related health outcomes including FBS, HbA_1c,_ and anthropometrics measurements were examined in 1 one-arm trial [57] and 6 randomized controlled trials [54,56,59,61-63]. All 7 studies reported improvement in the blood glucose levels (FBS or HbA_1c_ levels) of participants postintervention. However, these changes were not significant in 3 of these studies [54,56,57].

Of the 10 included studies, 3 (30%; one 1-arm trial [57] and 2 randomized controlled trials [60,62]) reported the effects of the interventions on anthropometric measures. However, in the 1-arm study by Ruggeiro et al [57], the mean BMI in the intervention group decreased significantly by 0.6 kg/m^2^. This reduction was attributed to improvements in the participants’ dietary choices. None of the studies reported improvements in the participants’ weight or other clinical indicators.

**Table 2:** Nutrition-related primary or secondary outcomes of the included studies.

Study	Study design	Outcome category	Results of the intervention group
Aligholipour et al [59], 2019	2-arm randomized controlled trial	Nutrition-related health outcome FBS^a^ level Nutrition-related cognitive or behavioral outcomes Dietary self-efficacy	Significant reduction in FBS level (−36.9; *P*<.001) Significant improvement in dietary self-efficacy (*P*<.001)
Arora et al [53], 2012	1-arm nonrandomized pilot trial	Nutrition-related cognitive or behavioral outcomes Dietary consumption Knowledge and skills	Significant increase in fruits and vegetables consumption by 26.5% of participants (*P*<.05) Nonsignificant change in diabetes knowledge score
Burner et al [54], 2017	2-arm randomized controlled trial	Nutrition-related health outcome HbA_1c_^b^ level Nutrition-related cognitive or behavioral outcomes Dietary self-efficacy	Nonsignificant reduction in HbA_1c_ level by −1.4% (*P*=.30) Nonsignificant change in dietary self-efficacy score (including general diet and specific diet)
Fortmann et al [62], 2017	2-arm randomized controlled trial	Nutrition-related health outcome HbA_1c_ level BMI Weight	Significant reduction in HbA_1c_ levels by −0.8% (*P*=.03) at 3 months and by −0.8% (*P*=.03) at 6 months postbaseline Nonsignificant change in BMI Nonsignificant change in weight
Moussa et al [58], 2013	2-arm randomized controlled trial	Nutrition-related cognitive or behavioral outcomes Knowledge and skills	Significant increase in meal planning score (*P*=.01)
Nelson et al [61], 2021	3-arm randomized controlled trial	Nutrition-related health outcome HbA_1c_ level Nutrition-related cognitive or behavioral outcomes PDQ^c^ score	Significant reduction in HbA_1c_ levels by −0.26% (*P*=.02) at 3 months and by −0.31% (*P*=.04) at 6 months postbaseline Nonsignificant change in PDQ
Porter et al [55], 2009	1-arm nonrandomized trial	Nutrition-related cognitive or behavioral outcomes Knowledge and skills	Significant improvement in diabetes-related knowledge by 39% (*P*<.05) Significant improvement in carbohydrate counting skill by 39% (*P*<.05) Significant improvement in ability to plan a meal plate by 31% (*P*<.05)
Robertson et al [56]; 2007	2-arm randomized controlled trial	Nutrition-related health outcome HbA_1c_ level Nutrition-related cognitive or behavioral outcomes Dietary consumption	Nonsignificant reduction in HbA_1c_ level (−0.8; *P*=.06) Nonsignificant change in total calorie intake Nonsignificant change in calories obtained from carbohydrates, proteins, or fats
Ruggiero et al [57]; 2014	1-arm nonrandomized trial	Nutrition-related health outcome HbA_1c_ level BMI Nutrition-related cognitive or behavioral outcomes Dietary self-efficacy	Nonsignificant reduction in HbA_1c_ level (−0.3; *P*=.053) Significant reduction in BMI level (−0.5, *P*=.02, at 3 months; and −0.7, *P*=.02, at 6 months) Nonsignificant change for general diet (*P*=.22) Nonsignificant change for general diet [61] (*P*=.09) Nonsignificant improvement in specific diet (*P*=.42) Nonsignificant change for specific diet rev (*P*=.91) Significant improvement in modifying meat (*P*=.01) Nonsignificant changes for avoiding fat (*P*=.70) Nonsignificant changes for diet subscale (*P*=.01)
Whittemore et al [60], 2020	2-arm randomized waitlist-controlled pilot trial	Nutrition-related health outcome HbA_1c_ level BMI Nutrition-related cognitive or behavioral outcomes Dietary self-efficacy	Significant reduction in HbA_1c_ level by −1.76% (*P*<.01) Nonsignificant change in BMI (*P*=.22) Significant improvement in dietary self-efficacy (*P*<.01)

^a^FBS: fasting blood sugar.

^b^HbA_1c_: hemoglobin A_1c_.

^c^PDQ: Personal Diabetes Questionnaire.

### Behavior Change Theories and Techniques

Of the 10 included studies, only 2 (20%) [57,60,63] described the use of behavioral change theories in designing interventions. The theories mentioned in these studies were the social cognitive theory, the health action process approach model, and the empowerment theory.

The studies included in this review used substantially heterogeneous terms to describe the intervention content and rarely reported their BCTs in detail, which limited the possibility of identifying their mechanisms of action. We used the BCT taxonomy [42] to develop a list of BCTs that appeared to be used according to the reported intervention content for each study. Table 3 summarizes the frequencies of the BCT clusters implemented across the studies in this review.

The coding of intervention strategies showed that 10 (62%) of the 16 BCT clusters were used across the included studies. *Shaping knowledge* (BCT 4; 10/10, 100% studies) and *natural consequences* (BCT 5; 9/10, 90% studies) were implemented in almost all studies, whereas *repetition and substitution* (BCT 8; 1/10, 10% study) and *regulation* (BCT 11; 1/10, 10% study) were only implemented in 1 study.

In addition to the 16 main behavior change clusters, intervention components can be further classified into 93 single BCTs [42]. A detailed summary of the BCTs included in each study is presented in Table 4.

The number of BCTs implemented across all the studies ranged from 2 [55] to 12 [63]. The most frequently implemented individual BCT was *instruction on how to perform the behavior* (BCT 4.1; 10/10, 100% studies), which was used across all studies. The second most frequently used individual BCT was *information about health consequences* (BCT 5.1; 9/10, 90% studies), which was implemented in 9 studies in this review [53-55,57,58]. The other common individual BCTs in the included studies were *credible source* (BCT 9.1; 8/10, 80% studies) and *social support (unspecified)* (BCT 3.1; 8/10, 80% studies).

**Table 3:** Studies implementing each BCT^a^ cluster (n=10).

Behavior change cluster	Studies, n (%)
4. Shaping knowledge^b^	10 (100)
5. Natural consequences	9 (90)
3. Social support	8 (80)
9. Comparison of outcomes	8 (80)
2. Feedback and monitoring	7 (70)
1. Goals and planning	4 (40)
10. Reward and threat	3 (30)
6. Comparison of behavior	2 (20)
8. Repetition and substitution	1 (10)
11. Regulation	1 (10)

^a^BCT: behavior change technique.

^b^Cluster numbers are based on the actual numbers used in the BCT taxonomy developed by Michie et al [42].

**Table 4:** Theoretical approaches, BCT^a^ clusters, and BCTs implemented across studies.

Study	Theoretical approach	Behavior change clusters^b^	BCTs^b^
Aligholipour et al [59], 2019	ND^c^	2. Feedback and monitoring 3. Social support 4. Shaping knowledge 5. Natural consequences 9. Comparison of outcomes	2.2. Feedback on behavior 2.7. Feedback on outcome of behavior 3.1. Social support (unspecified) 4.1. Instruction on how to perform the behavior 5.1. Information about health consequences 9.1. Credible source
Arora et al [53], 2012	ND	1. Goals and planning 3. Social support 4. Shaping knowledge 5. Natural consequences 9. Comparison of outcomes 10. Reward and threat	1.1. Goal setting (behavior) 1.4. Action planning 3.1. Social support (unspecified) 4.1. Instruction on how to perform the behavior 5.1. Information about health consequences 9.1. Credible source 9.2. Pros and cons 9.3. Comparative imagining of future outcomes 10.2. Material reward
Burner et al [54], 2017	ND	2. Feedback and monitoring 3. Social support 4. Shaping knowledge 5. Natural consequences 9. Comparison of outcomes 10. Reward and threat	2.2. Feedback on behavior 2.7. Feedback on outcome of behavior 3.1. Social support (unspecified) 3.3. Social support (emotional) 4.1. Instruction on how to perform the behavior 5.1. Information about health consequences 9.1. Credible source 10.4. Social reward
Fortmann et al [62], 2017	ND	2. Feedback and monitoring 3. Social support 4. Shaping knowledge 5. Natural consequences 9. Comparison of outcomes	2.3. Self-monitoring of behavior 3.1. Social support (unspecified) 4.1. Instruction on how to perform the behavior 5.1. Information about health consequences 9.1. Credible source
Moussa et al [58], 2013	ND	3. Social support 4. Shaping knowledge 5. Natural consequences 9. Comparison of outcomes	3.1. Social support (unspecified) 4.1. Instruction on how to perform the behavior 5.1. Information about health consequences 9.1. Credible source
Nelson et al [61], 2021	ND	1. Goals and planning 2. Feedback and monitoring 3. Social support 4. Shaping knowledge 5. Natural consequences 9. Comparison of outcomes	1.1. Goal setting (behavior) 2.2. Feedback on behavior 2.7. Feedback on outcome of behavior 3.1. Social support (unspecified) 3.3. Social support (emotional) 4.1. Instruction on how to perform the behavior 5.1. Information about health consequences 9.1. Credible source
Porter et al [55], 2009	ND	4. Shaping knowledge 5. Natural consequences	4.1. Instruction on how to perform the behavior 5.1. Information about health consequences
Robertson et al [56], 2007	ND	2. Feedback and monitoring 3. Social support 4. Shaping knowledge	2.2. Feedback on behavior 2.3. Self-monitoring of behavior 3.1. Social support (unspecified) 3.3. Social support (emotional) 4.1. Instruction on how to perform the behavior
Ruggiero et al [57], 2014	Social cognitive theory	1. Goals and planning 2. Feedback and monitoring 4. Shaping knowledge 5. Natural consequences 6. Comparison of behavior 8. Repetition and substitution 9. Comparison of outcomes 10. Reward and threat	1.1. Goal setting (behavior) 2.2. Feedback on behavior 4.1. Instruction on how to perform the behavior 5.1. Information about health consequences 6.1. Demonstration of the behavior 6.2. Social comparison 8.1. Behavioral practice and rehearsal 9.1. Credible source 9.2. Pros and cons 10.2. Material reward
Whittemore et al [60], 2020	HAPA^d^ model Empowerment theory Social cognitive theory	1. Goals and planning 2. Feedback and monitoring 3. Social support 4. Shaping knowledge 5. Natural consequences 6. Comparison of behavior 9. Comparison of outcomes 11. Regulation	1.1. Goal setting (behavior) 1.2. Problem-solving 1.3 Goal setting (outcome) 1.4. Action planning 2.3. Self-monitoring of behavior 3.1. Social support (unspecified) 4.1. Instruction on how to perform the behavior 5.1. Information about health consequences 6.1. Demonstration of the behavior 9.1. Credible source 9.2. Pros and cons 11.2. Reduce negative emotions

^a^BCT: behavior change technique.

^b^The clusters and BCT numbers are based on the actual numbers used in the BCT taxonomy developed by Michie et al [42].

^c^ND: none described.

^d^HAPA: health action process approach.

### Risk of Bias Across Studies

**Figure 2:**
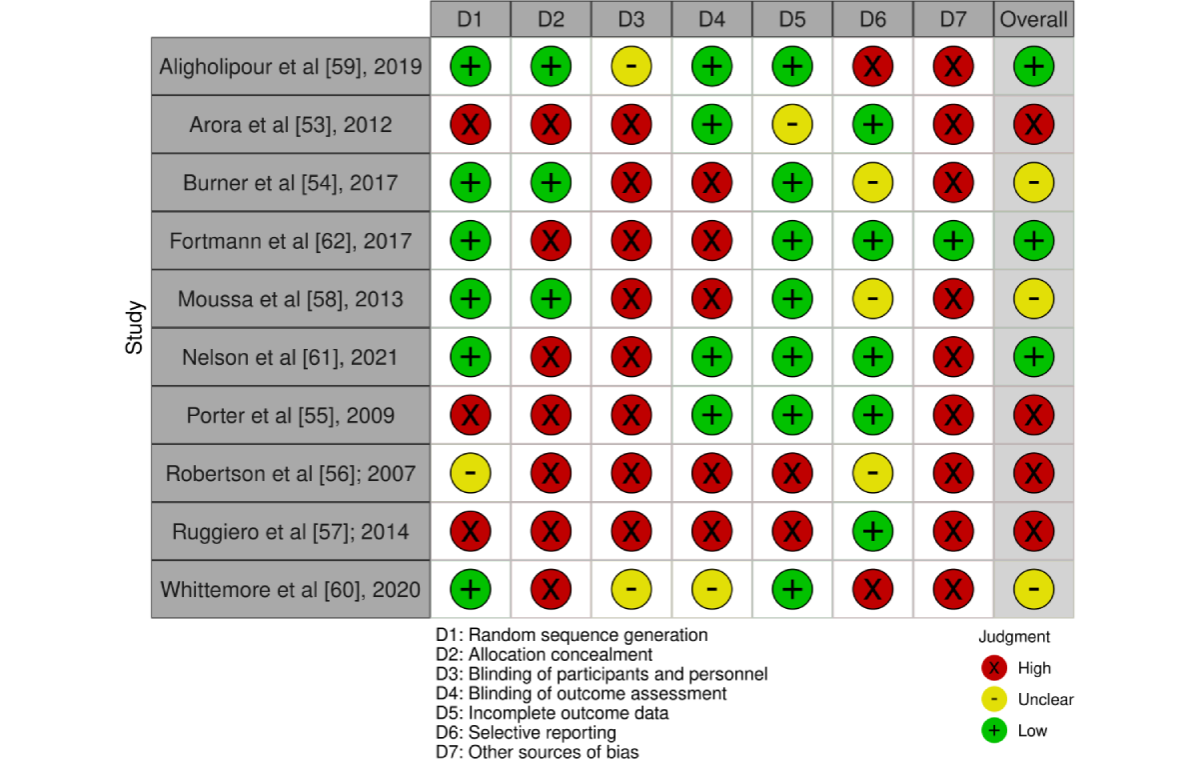
Risk-of-bias summary: review authors’ judgments about each risk-of-bias item for each included study.

**Figure 3:**
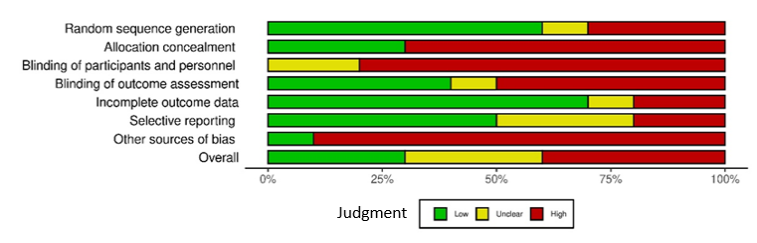
Risk-of-bias judgments about each risk-of-bias item presented as percentages across all included studies.

Details of the risk of bias of the included studies are summarized in Figures 2 and 3 [53-62], using the Cochrane Risk-of-Bias Assessment Tool (version 2) [65], and in Multimedia Appendix 2 [53-62].

### Allocation

Of the 10 studies included in this review, 6 (60%) had acceptable sequence generation [54,58-62], and 3 (30%) [53,55,57] were 1-arm nonrandomized controlled trials, and the sequence generation of participants was not possible with their design. These 3 studies were categorized as having a high risk of bias. In the study by Robertson et al [56], the method of generating random number sequences was not described, and this study was classified as having an unclear risk of bias.

A total of 3 (30%) [54,58,59] of the 10 studies in this review described an appropriate allocation concealment method. In these studies, the methods for allocation concealment were the use of a closed envelope [54,59] or a container, including equal *intervention* and *control* choices to draw [58]. In 4 (40%) of the 10 studies [56,60-62], the method used to conceal the allocation sequence was not provided. Hence, intervention allocations could have been foreseen before or during the enrollment. Another 3 studies [53,55,57] were 1-arm nonrandomized controlled trials, and the allocation concealment of participants was not possible with their design.

### Blinding

Blinding of participants is often not feasible in dietary behavior studies, and none of the studies in this review blinded the participants. Furthermore, there were no comments on blinding of program personnel in 4 studies [53,55,57,59] and outcome assessors in 6 studies [53,55,57,59-61]. However, in 3 of these studies [53,55,59], data were collected through surveys; therefore, blinding of assessors was not necessary, and the outcome measurement was not likely to be influenced by lack of blinding. These studies were assigned a low risk of bias.

### Incomplete Outcome Data

Of the 10 included studies, 7 (70%) [54,55,58-62] were assigned a low risk of bias. These studies either had 0 or minimal missing data (which was unlikely to have a great influence on outcome data) [55,58,59], or missing data were imputed using appropriate methods [54,60-62].

The study by Arora et al [53] had modest attrition (13%) by the 3-week assessment, but reasons for attrition were not provided, and analyses for handling incomplete data were not performed. Consequently, this study was identified as having an unclear risk of bias [53].

The remaining 2 studies failed to provide complete outcome data and had a high risk of bias [56,57]. In 1 of these studies [57], a series of mixed-effects analyses with time as a single repeated measures factor was performed to address missing data; however, as the attrition rate was high and differences in characteristics between completers and noncompleters were not provided, we felt that the results were at a high risk of attrition bias. The study [56] had incomplete demographic data, and consequently, group differences between completers and noncompleters were not reported accurately.

### Selective Reporting

We were able to identify a published protocol for 4 (40%) of the 10 included studies [59-62]. In 2 of these studies [61,62], the reported outcomes matched those reported in the registered protocol; therefore, these studies were considered to have a low risk of bias.

### Other Potential Sources of Bias

We did not observe any strong source of other bias in 1 (10%) [62] of the 10 studies included in this review. However, other studies had high [53,54,56-58] or unclear [55] risks of having other biases. Studies were judged to have a high risk of bias because of having ungeneralizable data [53,59], small sample sizes without formal power calculations or convenience sampling [54,55,58], using unvalidated surveys [58], providing monetary incentives [57,60,61], recruiting participants who owned mobile phones only [53,54,59], not providing sufficient demographic data [56], and having a potentially active intervention for the control group [54,58,59].

## Discussion

### Principal Findings

This review advances the existing evidence based on previous reviews [25,26,31] by assessing studies that focused on nutritional behaviors and nutrition-related health outcomes and were conducted among socioeconomically disadvantaged people with T2D. In this systematic review, although 10 studies (including 947 participants) were identified, smaller subsets assessed particular outcomes, and both methods and findings were somewhat mixed; therefore, evidence remains limited but is suggestive of the potential of digitally delivered nutrition interventions in socioeconomically disadvantaged people with T2D. Studies in this review included controlled and noncontrolled interventions covering mobile- and web-based interventions, ranging from 1 day to 1 year in duration, with or without health coaching, among individuals aged 45.4 to 55.8 years from disadvantaged population groups.

### Effect of the Interventions on Nutrition Behavior Outcomes

Studies comparing digitally delivered interventions versus controls and pre‐ and posttest comparisons yielded broadly positive results. However, considerable diversity was observed in the range of outcomes measured.

A total of 9 studies in this review, including 3 one-arm trials [53,55,57] and 6 randomized controlled trials [54,56,58,60,61], reported nutrition-related cognitive or behavioral outcomes. Of the 3 one-arm trials, all showed improvements in nutritional behavior outcomes, and 1 specifically provided evidence regarding healthier food choices, such as increased consumption of fruits and vegetables, among participants [53]. By contrast, of the 6 controlled trials, only 2 provided significant improvement in nutritional behaviors [58,59]. All 9 studies showed positive effects of interventions on nutritional knowledge, skills, and self-efficacy, which are the key mediators of healthy eating. Given the rapid increase in the disease burden and prevalence of diabetes worldwide, and especially in disadvantaged populations, it is notable that the weight of evidence suggests that these digitally delivered interventions can have a positive effect on dietary choices and engagement in healthy eating behaviors or on their cognitive or behavioral determinants among socioeconomically disadvantaged populations.

Differences across nutritional outcomes might reflect the heterogeneous dietary recommendations provided to participants in the interventions or the type and amount of tailoring of the materials. It appears that interventions that incorporated feedback and suggestions from members of ethnic communities and provided culturally appropriate materials (eg, cooking lessons or tailored nutritional education or counseling) resulted in better outcomes [55-57,60,62]. Another explanation for the mixed results may be intervention complexity or dose, as interventions varied in terms of the number of components, quantity of materials, and support provided to participants. In this review, interventions with fewer elements and simple design characteristics to address the needs of people with low literacy and electronic literacy [55,58] reported more positive outcomes and higher retention rates than those with more complex designs [56,57]. Previous systematic reviews have also indicated that overly complex interventions may lead to a lack of motivation owing to confusion, provision of irrelevant content, and technical difficulties [66,67]. Furthermore, the studies included in this review used substantially different nutrition assessment methods, which were often study specific. This diversity in methods for assessing dietary intake might have also contributed to the observed heterogeneity in the results.

Finally, based on the results observed in 7 randomized controlled trials and 3 one-arm trials, the overall effectiveness of the interventions did not seem to vary according to whether the interventions were fully automated or whether they were combined with additional intervention components such as the presence of an educator. This reinforces the idea that automated programs, with their potential to save labor, could be seen as a way to deliver diabetes interventions efficiently and effectively. Such programs can also increase the predictability of outcomes. When people follow a standardized care path supported by automation, they are more likely to stay on track toward the accepted outcomes [31]. Moreover, automation can assist in detecting when a patient deviates from the recommended care plan, enabling the health care team to intervene at the right moment [28,29,31].

### Effect of the Interventions on Nutrition-Related Health Outcomes

This review also provided some evidence that improvement in blood glucose levels can be achieved by using digitally delivered interventions for people from socioeconomically disadvantaged backgrounds.

A total of 7 studies examined changes in blood glucose levels (FBS or HbA_1c_), of which 4 [59-62] revealed significant improvement. In these 4 studies, decreases in HbA_1c_ levels ranged between 0.3% [53] and 1.8% [60], and FBS decreased by 36.9 mmol/L [59]. The other 3 studies [54,56,57] reported positive but nonstatistically significant improvements.

This range of changes in HbA_1c_ levels is somewhat similar to that reported in in-person and digitally delivered T2D self-management education interventions in general (not specifically disadvantaged) [68,69] and disadvantaged populations [70]. For instance, web-based programs have been shown to decrease HbA_1c_ levels by 0.47% to 1.49%, mobile apps by 0.4% to 1.9% [71], other mobile-based programs by an average of 0.8% [72], and telehealth interventions by an average of 0.17% [73]. In addition, in socioeconomically disadvantaged people with T2D, a previous systematic review and meta-analysis on the effects of digitally delivered T2D self-management education interventions on glycemic control found modest but clinically significant reductions in HbA_1c_ levels, ranging from 0.2% to 0.8% at 6 months. [26]. Although the observed changes in HbA_1c_ levels are rather small across all types of studies, these changes are clinically meaningful, as previous research has demonstrated that even a 1% decrease in HbA_1c_ levels for 10 years is related to a 37% reduction in microvascular complications and 21% reduction in morbidity from diabetes [74].

### BCTs Used by the Interventions

Despite previous evidence that theoretically grounded interventions are more effective than atheoretical approaches, 8 (80%) of the 10 studies did not report a theoretical underpinning. Those that did [57,63] reported significant intervention effects for some but not all outcomes. Further work is needed to examine the most appropriate evidence-based theoretical frameworks for developing intervention content for these target groups.

There were considerable variations in the type and number of BCTs included in the interventions. On average, 5 (31%) of the 16 different BCT clusters were implemented in each study. The BCT clusters that formed the central *building blocks* of interventions in this review were providing information, informing natural consequences, providing social support, delivering feedback and self-monitoring, and setting goals. These BCT clusters are similar to those incorporated in successful in-person individual and group‐based interventions [75] or those reported in reviews of digitally delivered interventions [22,23,40,75-78].

The number of single BCTs implemented in the interventions varied from 2 to 12 across studies. Although in studies with a minimum number of BCTs, significant improvements in healthy eating and diet-related health outcomes were reported [55], several studies included >6 BCTs and yet showed no significant outcomes. Therefore, these results do not support the previously suggested associations between greater intervention effectiveness and increasing number of BCTs [75,77-79]. A similar result was reported in a review by Villinger et al [27], which revealed no association between the number of BCTs and the efficacy of app-based mobile interventions on nutritional behaviors. However, it should be noted that most studies did not explicitly report on BCTs; hence, they were inferred by the review authors based on the intervention content described.

The most frequently identified single BCTs were *instruction on how to perform the behavior*, *information about health consequences*, and *social support*. These results suggest that researchers rely heavily on the assumption that providing instructions on how to perform the behavior and information about health consequences are the most effective tools for bringing about changes in people’s healthy behaviors. However, evidence shows that health behaviors are motivated by a broader range of perceived benefits than health considerations alone [80]. Moreover, social support provided in the included interventions mainly focused on encouragement and counseling from HCPs or family supporters, and mentions of practical support were not present, although evidence suggests that such sources of social support are also beneficial for improving healthy behaviors and clinical outcomes [81]. There were no similar reviews that have reported on BCTs used in digitally delivered nutritional support interventions. However, the most commonly used BCT categories identified in this review correspond closely with those found in a previous review by Seppälä et al [81] that identified targets, mediators, and change strategies for physical activity and nutrition behavior changes in Finnish policy papers on workplace health promotion.

Although we acknowledge that the taxonomy of BCTs is comprehensive and not all techniques may be needed to change nutrition behavior, among the 93 BCTs proposed in the taxonomy by Michie et al [42], only 19 were used in the interventions. These findings suggest the need for researchers to explore the potential of not commonly used BCTs, but others, such as problem-solving, that may contribute to increasing the effectiveness of interventions to promote nutritional behaviors.

### Tailoring of Interventions to Target Population

In this review, tailoring the digital design using digital elements or language suitable for people with low literacy or electronic literacy [53,54] and targeting intervention content based on the cultural background and language of intended participants comprised the main forms of intervention tailoring [55-57,60,62]. These results are in line with the results of previous systematic reviews on the effectiveness of self-management support interventions in patients with diabetes or other chronic conditions [3,22,26], which reported that the most common ways of targeting the intervention to the population group interventions are by (1) modifying content to culture or language, (2) using personnel familiar with study participants (eg, community health workers, health educators, and nurses), or (3) individually tailoring an intervention to self-reported participants’ data (eg, blood glucose level, diet and physical activity data, diabetes barriers, social support, and psychosocial challenges).

Although tailoring the content to language has been shown to be helpful in some studies [3,25,55,58], for maximum effectiveness, researchers should also consider participants’ literacy and numeracy levels in the design of digital interventions for people who are disadvantaged. Low literacy is a prevalent barrier to understanding and acting on health information among these populations [26]. People with lower levels of literacy and numeracy often struggle with navigating web-based portals and understanding and trusting web-based health information and prefer to rely on verbal communication about their health-related issues [82,83]. Some studies that factored in electronic literacy considerations suggested that this led to an increased understanding of educational content by end users [83].

### Strengths and Limitations

To the best of our knowledge, this is the first review to evaluate the effect of digitally delivered interventions on nutrition-related cognitive or behavioral outcomes and nutrition-related health outcomes among socioeconomically disadvantaged people with T2D. A comprehensive search strategy was developed in consultation with an expert librarian, and the search was performed using 7 electronic databases from medical and nonmedical sources. Furthermore, we used a reliable BCT taxonomy [42] to develop a list of effective BCTs for each study. This helped us to handle heterogeneity across multicomponent and complex interventions and to unravel the effects of intervention components on nutrition behavior outcomes. However, a potential limitation is that, given the limited reporting of these constructs, not all BCTs may have been captured accurately or at all.

Our search was limited to English language studies, and only published peer review studies were included. Owing to the multifaceted and complex nature of digitally delivered interventions, there were a multitude of primary and secondary outcomes for which data were extracted; however, these were specified a priori, and we have only reported on the outcomes listed in the registered protocol. The findings should also be interpreted with some caution, given the limited number of studies in each group of technologies and in general. Comparable to similar reviews, the methodological quality of studies varied; for example, almost none of the studies blinded outcome assessors or participants to the treatment group. However, blinding participants in behavioral interventions is difficult, and this criticism is common in many similar reviews. In addition, most studies were conducted in the United States, limiting the generalizability to low- to middle-income countries. Given that three-quarters of the world’s diabetes population lives in these countries, more digitally delivered interventions should be developed and tested for people with diabetes in low- to middle-income countries. Such interventions must be properly contextualized and adapted to the culture, language, literacy and numeracy skills, health systems, and access to the internet and digital devices of the populations in these countries.

Another limitation of this review was that definitions of and thresholds for belonging to disadvantaged populations differed to some extent between studies, revealing that there is no one agreed-on *cut-off* for this. We specified that the terms related to SES, low income, or minority had to be used in the title or body of the paper to refer to participants for studies to be included, and we searched an extensive range of concepts to target low SES, such as residential areas, belonging to a health clinic serving disadvantaged groups, belonging to certain ethnic groups, and indicators of income. Thus, we captured studies considering disadvantage in different ways. However, relevant papers that did not use these terms may have been missed.

One challenge in answering our research question was that although it is well known that a healthy diet plays an important role in managing diabetes, several included interventions did not have a main focus on nutrition but included nutrition education as part of their multicomponent interventions to improve T2D self-management. Participants might have responded more effectively to interventions focused intensively on 1 area of behavior change (such as nutrition) at a time rather than multicomponent interventions [27]. This highlights a further gap in the current literature that needs to be addressed in future studies.

### Conclusions

Despite research highlighting the serious need for effective behavior change support for people from susceptible populations to assist in reducing health inequities, relatively few studies of digital interventions have included these populations or particularly tailored materials to fit their cultural, linguistic, literacy, numeracy, or other needs.

Owing to the limited evidence base, few claims can be made regarding the effectiveness of digitally delivered interventions on nutrition behavior outcomes for disadvantaged people with T2D. However, we found some support for the efficacy of these interventions on healthy behaviors among disadvantaged populations, which is an essential prerequisite for changes in the clinical metabolic parameters. Our review showed small but positive changes in some eating behaviors and related health outcomes such as HbA_1c_ levels, although the results were highly heterogeneous. Heterogeneity is likely because of variations in the study design (eg, comparator groups and technologies used) and methodological quality (eg, instruments to measure outcomes assessed). Differences in the intervention content, including the BCTs used, is also a possible contributing factor.

In summary, a small body of evidence suggests that digital interventions can influence eating behaviors among certain disadvantaged groups with T2D. If this effect is generalized on a population, it could potentially have a considerable impact on public health. Specifically, digital technology can be used to meet the specific needs of disadvantaged patients for T2D self-management with regard to eLiteracy, literacy, numeracy, and cultural considerations. Further evidence is needed regarding the specific features of effective digital interventions for supporting healthy behaviors in the most disadvantaged people and helping address health inequities.

## Appendix

Multimedia Appendix 1 Search strategies for the MEDLINE complete, CINAHL complete, Global Health, and Applied Science databases.

Multimedia Appendix 2 Checklist for the quality assessment of studies.

## Abbreviations

BCT: behavior change technique
DSM: diabetes self-management
FBS: fasting blood sugar
HbA_1c_: hemoglobin A1C
HCP: health care provider
MMS: multimedia messaging service
PICO: population, intervention, control, and outcomes
PRISMA: Preferred Reporting Items for Systematic Reviews and Meta-Analyses
SDSCA: Summary of Diabetes Self-Care Activities
SES: socioeconomic status
T2D: type 2 diabetes
