# Supplementary material for: Digitally Delivered Interventions to Improve Nutrition Behaviors Among Resource-Poor and Ethnic Minority Groups With Type 2 Diabetes: Systematic Review
Source: J Med Internet Res. 2024 Feb 1;26:e42595. doi: 10.2196/42595 (PMC10870209; doi:10.2196/42595)
Supplement: Multimedia Appendix 1 [file jmir_v26i1e42595_app1.docx]

**Multimedia Appendix 1: Search strategies for “Medline complete”, “Cinahl complete”, “Global health”, and “Applied science” data bases**

|  | Search options |
| --- | --- |
|  |  |
| S97 | S93 AND S94 AND S95 AND S96 |
| S96 | S1 OR S2 OR S3 OR S4 OR S5 OR S6 OR S7 OR S8 OR S9 OR S10 OR S11 OR S12 OR S13 OR S14 OR S15 OR S16 OR S17 OR S18 OR S19 OR S20 OR S21 OR S22 OR S23 OR S24 OR S25 OR S26 OR S27 OR S28 OR S29 OR S30 OR S31 OR S32 OR S33 OR S34 OR S35 OR S36 OR S37 OR S38 OR S39 OR S40 OR S41 OR S42 OR S43 OR S44 OR S45 OR S46 |
| S95 | S47 OR S48 OR S49 OR S50 OR S51 OR S52 OR S53 OR S54 OR S55 OR S56 OR S57 OR S58 OR S59 OR S60 OR S61 OR S62 OR S63 OR S64 OR S65 OR S66 OR S67 OR S68 OR S69 OR S70 OR S71 OR S72 OR S73 OR S74 |
| S94 | S75 OR S76 OR S77 OR S78 OR S79 OR S80 OR S81 OR S82 OR S83 OR S84 OR S85 OR S86 |
| S93 | S87 OR S88 OR S89 OR S90 OR S91 OR S92 |
| **DIABETES** |  |
| S88 | (MM "Diabetes Mellitus, Type 2") OR (MM "Diabetes Mellitus") |
| S92 | (MM "Insulin Resistance") |
| S91 | AB "insulin resistance" OR TI "insulin resistance" |
| S87 | AB Diabet* OR TI Diabet* |
| S90 | AB T2D OR TI T2D |
| S89 | AB T2DM OR TI T2DM |
| **HEALTHY EATING** |  |
| S84 | (MH "Dietary Carbohydrates") OR (MH "Carbohydrates") |
| S86 | (MH "Feeding Behavior") |
| S77 | (MM "Diet") OR (MH "Healthy Diet") |
| S79 | (MM "Food+") OR (MM "Diet, Food, and Nutrition+") |
| S83 | AB Carbohydrate* OR TI Carbohydrate* |
| S76 | AB Diet* OR TI Diet* |
| S78 | AB Eat* OR TI Eat* |
| S85 | AB Feed* OR TI Feed* |
| S80 | AB Food* OR TI Food* |
| S81 | AB Fruit* OR TI Fruit* |
| S75 | AB Nutritio* OR TI Nutrio* |
| S82 | AB Vegetable* OR TI Vegetable* |
| **SOCIOECONOMIC POSITION** |  |
| S69 | (MH "Ethnic Groups") |
| S58 | (MH "Poverty Areas") |
| S67 | (MH "Vulnerable Populations") OR (MH "Medically Underserved Area") |
| S72 | (MM "Minority Groups") |
| S56 | (MM "Poverty") |
| S48 | AB "low* socio economic* position*" OR TI "low* socio economic* position*" |
| S49 | AB "low* socio-economic* position*" OR TI "low* socio-economic* position*" |
| S47 | AB "low* socioeconomic* position*" OR TI "low* socioeconomic* position*" |
| S53 | AB "Low-SEP" OR TI "Low-SEP" |
| S52 | AB "Low-SES" OR TI "Low-SES" |
| S50 | AB "L-SEP" OR TI "L-SEP" |
| S51 | AB "L-SES" OR TI "L-SES" |
| S65 | AB “low* Income*” OR TI “low* Income*” |
| S66 | AB “low* middle class*” OR TI “low* middle class*” |
| S63 | AB “low* socio economic* class*” OR TI “low* socio economic* class*” |
| S60 | AB “low* socio economic* status” OR TI “low* socio economic* status” |
| S64 | AB “low* socio-economic* class*” OR TI “low* socio-economic* class*” |
| S62 | AB “low* socioeconomic* class*” OR TI “low* socioeconomic* class*” |
| S61 | AB “low* socio-economic* status” OR TI “low* socio-economic* status” |
| S59 | AB “low* socioeconomic* status” OR TI “low* socioeconomic* status” |
| S73 | AB Disadvantage* OR TI Disadvantage* |
| S70 | AB Ethnic* OR TI Ethnic* |
| S71 | AB Minorit* OR TI Minorit* |
| S57 | AB Povert* OR TI Povert* |
| S54 | AB SEP OR TI SEP |
| S55 | AB SES OR TI SES |
| S68 | AB Underserved OR TI Underserved |
| S74 | AB Vulnerable* OR TI Vulnerable* |
| **m/e HEALTH** |  |
| S44 | (MH "Cell Phone Use") |
| S14 | (MH "Health Information Interoperability") OR (MH "Health Information Systems") |
| S12 | (MH "Internet+") |
| S31 | (MM "Blog") |
| S23 | (MM "Computers") OR (MM "computer based") |
| S37 | (MM "Information Technology") |
| S29 | (MM "Internet") OR (MH "Internet Access") OR (MH "Patient Portals") |
| S24 | (MM "Online Systems") |
| S11 | (MM "Online Systems") OR (MM "Online Social Networking") |
| S26 | (MM "Smartphone") OR (MM "Cell Phone") |
| S6 | (MM "Social Media") OR (MM "Text Messaging") |
| S19 | (MM "Software") |
| S15 | (MM "Technology+") OR (MH "Educational Technology+") |
| S35 | (MM "Telemedicine") |
| S30 | (MM "Web Browser") OR (MM "web based") |
| S45 | AB "Cell Phone*" OR TI "Cell Phone*" |
| S34 | AB "e-Health" OR TI "e-Health" |
| S21 | AB "e-learning" OR TI "e-learning" |
| S22 | AB "Electronic Health" OR TI "Electronic Health" |
| S1 | AB "Health* Information*" OR TI "Health* Information*" |
| S3 | AB "mHealth" or TI "mHealth" |
| S4 | AB "mobile health" or TI "mobile health" |
| S25 | AB "mobile learning" OR TI "mobile learning" |
| S9 | AB "short message* service*" or TI "short message* service*" |
| S28 | AB "Smart phone*" OR TI "Smart phone*" |
| S38 | AB "Social Media" OR TI "Social Media" |
| S8 | AB "social media" or TI "social media" |
| S7 | AB "text messag*" or TI "text messag*" |
| S17 | AB APP OR TI APP |
| S18 | AB Application* OR TI Application* |
| S41 | AB APPs OR TI APPs |
| S32 | AB Blog* OR TI Blog* |
| S13 | AB computer* or TI computer* |
| S33 | AB eHealth OR TI eHealth |
| S43 | AB Internet* OR TI Internet* |
| S42 | AB Mobile* OR TI Mobile* |
| S39 | AB Online* OR TI Online* |
| S46 | AB Portal*" OR TI Portal* |
| S27 | AB Smartphone* OR TI Smartphone* |
| S10 | AB SMS or TI SMS |
| S20 | AB Software* OR TI Software* |
| S16 | AB technolog* OR TI technolog* |
| S5 | AB telehealth or TI telehealth |
| S36 | AB Telemedicine OR TI Telemedicine |
| S40 | AB Web* OR TI Web* |
| S2 | mHealth |
