# Supplementary material for: Digitally Delivered Interventions to Improve Nutrition Behaviors Among Resource-Poor and Ethnic Minority Groups With Type 2 Diabetes: Systematic Review
Source: J Med Internet Res. 2024 Feb 1;26:e42595. doi: 10.2196/42595 (PMC10870209; doi:10.2196/42595)
Supplement: Multimedia Appendix 2 [file jmir_v26i1e42595_app2.docx]

**Multimedia Appendix 2: Check list for studies’ quality assessment**

|  | **Quality Assessment** | | | | | |
| --- | --- | --- | --- | --- | --- | --- |
|  |  | | | | | |
|  | **Study** | **High** | **Low** | **Unclear** | **Support for judgement** | **Judgment comment** |
| **Sequence generation** | Aligholipour et al [59], 2019 |  | X |  | “Each pair of them (participants) randomly assigned to the MMS-based education or the in-person education groups in categories of four persons. Sequentially numbered, sealed, opaque envelopes were used to allocation concealment.” | -Simply describes as ‘sequentially numbered’ and allocated randomly  -The investigators described a random component in the sequence generation process.  -The method used to generate the allocation sequence was not described in full detail. However, it mentioned that participants were randomly assigned to the intervention group or the usual care group and the fact that they used Sequentially numbered, sealed envelopes. |
|  | Arora et al [53], 2012 | X |  |  | “Prospective cohort study (non-randomized trial)” | -No sequence generation of participants possible with this one arm study. |
|  | Burner et al [54], 2017 |  | X |  | “Parallel, randomized control trial with a 1:1 allocation”  “Once baseline assessment was complete, patients were randomized to receive the intervention condition (TExT-MED+FANS intervention) or control condition (TExT-MED with un-augmented social support and a pamphlet for supporters) by sequential closed envelope assignment. Envelopes were created before study initiation, and they were opened at baseline assessment by the RA enrolling the patient.” | -The investigators described a random component in the sequence generation process |
|  | Fortmann et al [62], 2017 |  | X |  | “Blocked random assignment with equal allocation was used to assign participants to Dulce Digital or usual care (UC), using a randomly generated numbers sequence. Participants were informed of group assignment after the baseline assessment” | -The investigators described a random component in the sequence generation process |
|  | Moussa et al [58], 2013 |  | X |  | “Forty-six consenting African American participants with type 1 or type 2 diabetes were randomly assigned to receive either the e-health intervention, eCare We Care (intervention group, n = 23) or the paper-based, text-only diabetes tutorials (comparison group, n = 23). To minimize selection bias, participants were asked to draw from a container that included 23 ‘electronic’ and 23 ‘paper’ choices as they arrived to attend their first educational session.” | -The investigators described a random component in the sequence generation process |
|  | Nelson et al [61], 2021 |  | X |  | “After enrolment, our team’s statistician used R software (v.3.5.1) to randomize participants (details in Supplementary Material) using optimal multivariate matching to ensure a covariate balance across study conditions (21). Participants were randomized to control or intervention arms; then those randomized to intervention were matched and randomized to REACH only or REACH with FAMSfor a 2:1:1 design.” | -The investigators described a random component in the sequence generation process. |
|  | Porter et al [55], 2009 | X |  |  | “One arm study” | -No sequence generation of participants possible with this one arm study. |
|  | Robertson et al [56], 2007 |  |  | X | “Thirty-three individuals with clinically diagnosed type 2 diabetes living on a Northern Plains Indian Reservation were recruited through advertisements and word-of-mouth to join a 6-month Internet-based intervention with the goal of controlling type 2 diabetes through lifestyle modifications.”  “Nineteen members of the same tribe with clinically diagnosed type 2 diabetes living on the same Northern Plains Indian Reservation were recruited as a non-intervention control group in the same manner as the intervention group.” | -Method used to generate the allocation sequence was not provided. |
|  | Ruggiero et al [57], 2014 | X |  |  | “Single-group repeated measures design”  “The participants were recruited from a university-based primary care clinic”.  “An initial run-in phase was conducted to serve multiple purposes, including to confirm that potentially eligible participants had basic skills in using the computer and SL; to ensure that individuals were committed to participation; and to gather feedback to further refine the intervention and support usage.” | -No sequence generation of participants possible with this single group study. |
|  | Whittemore et al [60], 2020 |  | X |  | “Upon completion of baseline data collection, eligible participants were randomly allocated to 1 of the 2 treatment conditions in a 1:1 allocation ratio using a computer-generated, block randomization procedure.” | -The investigators described a random component in the sequence generation process |
| **Allocation concealment** | Aligholipour et al [59], 2019 |  | X |  | “Opaque envelopes were used to allocation concealment”  “The patients and the nurses of the study setting were unaware to the comparison groups, the intervention details, the post-test time, and the study other details.” | -Concealment of allocation prior to assignment of intervention. |
|  | Arora et al [53], 2012 | X |  |  | “Prospective cohort study (non-randomized trial)” | -No allocation concealment with this study design; prospective cohort study. |
|  | Burner et al [54], 2017 |  | X |  | “Envelopes were created before study initiation, and they were opened at baseline assessment by the RA enrolling the patient.” | -Participants and investigators enrolling participants could not foresee assignment because envelopes were created before study initiation, and they were opened at baseline assessment by the RA enrolling the patient |
|  | Fortmann et al [62], 2017 | X |  |  | “Participants were informed of group assignment after the baseline assessment”” | -Method used to conceal the allocation sequence was not provided. Hence, intervention allocations could have been foreseen in advance of, during, enrolment. |
|  | Moussa et al [58], 2013 |  | X |  | “To minimize selection bias, participants were asked to draw from a container that included 23 ‘electronic’ and 23 ‘paper’ choices as they arrived to attend their first educational session.”  ” As group choices were selected, participants were assigned to separate ‘electronic’ or ‘paper’ instruction group classrooms, and they remained in each instruction group for the entire study.” | -Concealment of allocation prior to assignment of intervention. |
|  | Nelson et al [61], 2021 | X |  |  |  | -Method used to conceal the allocation sequence was not provided.  Hence, intervention allocations could have been foreseen in advance of, during, enrolment. |
|  | Porter et al [55], 2009 | X |  |  | “One arm study” | -No allocation concealment with this one arm study. |
|  | Robertson et al [56], 2007 | X |  |  | “Thirty-three individuals with clinically diagnosed type 2 diabetes living on a Northern Plains Indian Reservation were recruited through advertisements and word-of-mouth to join a 6-month Internet-based intervention with the goal of controlling type 2 diabetes through lifestyle modifications.”  “Nineteen members of the same tribe with clinically diagnosed type 2 diabetes living on the same Northern Plains Indian Reservation were recruited as a non-intervention control group in the same manner as the intervention group.”  “Participants were assigned a study ID to maintain confidentiality.” | -Method used to conceal the allocation sequence was not provided.  -Hence, intervention allocations could have been foreseen in advance of, during, enrolment. |
|  | Ruggiero et al [57], 2014 | X |  |  | “Single-group repeated measures design”  “The participants were recruited from a university-based primary care clinic”.  “An initial run-in phase was conducted to serve multiple purposes, including to confirm that potentially eligible participants had basic skills in using the computer and SL; to ensure that individuals were committed to participation; and to gather feedback to further refine the intervention and support usage.” | -No sequence generation of participants possible with this single group study. |
|  | Whittemore et al [60], 2020 | X |  |  | “At the end of the baseline data collection, an RA not involved in data collection told each participant their group assignment. Data collectors were blinded to treatment group assignment.” | -Method used to conceal the allocation sequence was not provided.  Hence, intervention allocations could have been foreseen in advance of, during, enrolment. |
| **Blinding of participants and personnel** | Aligholipour et al [59], 2019 |  |  | X |  | -Blinding of participants were not possible.  -Blinding of personnel has not mentioned. |
|  | Arora et al [53], 2012 | X |  |  | “Prospective cohort study (non-randomized trial)” | -No blinding of participants and personnel possible with this prospective cohort study design. |
|  | Burner et al [54], 2017 | X |  |  | “This is a parallel, nonblinded, randomized control trial with a 1:1 allocation.” | -Non-blinded study |
|  | Fortmann et al [62], 2017 | X |  |  | “The intervention was tested using a parallel-groups, nonblinded, randomized design” | -Non-blinded study |
|  | Moussa et al [58], 2013 | X |  |  | “Non blinded study” | -Non-blinded study |
|  | Nelson et al [61], 2021 | X |  |  | “After randomization, RAs called participants to explain their assigned condition” | -No blinding of participants. |
|  | Porter et al [55], 2009 | X |  |  |  | -No blinding of participants possible with this one arm study. |
|  | Robertson et al [56], 2007 | X |  |  | “Patients and personnel were not blinded to group allocation.” | -Non-blinded |
|  | Ruggiero et al [57], 2014 | X |  |  | “Single-group repeated measures design” | -No blinding of participants and personnel possible with this single-group repeated measures design |
|  | Whittemore et al [60], 2020 | X |  | X | “Data collectors were blinded to treatment group assignment.” | -Blinding of participants were not possible.  Stated data collectors blinded to treatment assignment |
| Blinding of outcome assessors | Aligholipour et al [59], 2019 |  | X |  |  | -No information provided regarding blinding of assessors. However, Collection of data was through surveys, so blinding of “assessors” was not necessary, and the outcome measurement was not likely to be influenced by lack of blinding. |
|  | Arora et al [53], 2012 |  | X |  |  | -No information provided regarding blinding of outcome assessor. However, Collection of data was through surveys, so blinding of “assessors” was not necessary and the outcome measurement was not likely to be influenced by lack of blinding. |
|  | Burner et al [54], 2017 | X |  |  | “Non-blinded RCT” | -Non-blinded |
|  | Fortmann et al [62], 2017 | X |  |  | “Non-blinded” | -Non-blinded |
|  | Moussa et al [58], 2013 | X |  |  | “Non-blinded” | -Non-blinded |
|  | Nelson et al [61], 2021 |  | X |  |  | -No information provided regarding blinding of outcome assessor. However, Collection of part of the data was through surveys, so blinding of “assessors” was not necessary and the outcome measurement was not likely to be influenced by lack of blinding. Other outcomes were HbA1c which needs clinical tests and again it is not likely to be influenced by lack of blinding. |
|  | Porter et al [55], 2009 |  | X |  |  | -No information provided about blinding of assessors. However, Collection of data was through surveys, so blinding of “assessors” was not necessary and the outcome measurement was not likely to be influenced by lack of blinding. |
|  | Robertson et al [56], 2007 | X |  |  | “Patients and personnel were not blinded” | -Non-blinded |
|  | Ruggiero et al [57], 2014 | X |  |  | “Self-report measures, BMI, and A1C were collected at baseline, 3 months (mid program), and 6 months (end of intervention) by trained research staff”. | -Blinding of outcome assessors to intervention allocation not possible with this single-group study design.  -Collection of part of the data was through surveys, so blinding of “assessors” was not necessary and the outcome measurement was not likely to be influenced by lack of blinding. Another outcome was HbA1c which needs clinical tests and again it is not likely to be influenced by lack of blinding. However there is chance of assessor bias in evaluating BMI. |
|  | Whittemore et al [60], 2020 |  |  | X |  | -Collection of part of the data was through surveys, so blinding of “assessors” was not necessary and the outcome measurement was not likely to be influenced by lack of blinding.  -There is chance of assessor bias in evaluating BMI. |
| Incomplete outcome data | Aligholipour et al [59], 2019 |  | X |  | “During the educational intervention, 2 patients from MMS-based education group and 1 person in the in-person education group were excluded from the study due to hospitalization. Therefore, the data retrieved from 63 patients were included in the final analysis” | -Reasons for attrition/exclusions were reported.  -Proportion of participants who were excluded is low and it is unlikely to have a great influence on outcome data. |
|  | Arora et al [53], 2012 |  |  | X | “At the end of the 3-week trial, 3 of the 23 (13.0%) subjects did not return to complete the exit interview and follow-up questionnaire.” | -Attrition was modest (13%) by the 3-week assessment.  -Reasons for attrition/exclusions were not reported, and analyses for handing incomplete data was not performed. (However, the proportion of participants who did not complete the exit interview (3/23) is unlikely to have a great influence on outcome data) |
|  | Burner et al [54], 2017 |  | X |  | “At the conclusion of the study, 82% (36/44) of patients followed up at 3 months. We were able to follow up with 60% (26/44) of supporters.”  “We found no differences in baseline characteristics for patients who completed follow-up versus those who did not,  nor in preliminary outcome measures for patients whose supporters received the messages versus those who did not.” | -Missing data have been imputed using appropriate methods.  -Acceptability: No patients or supporters dropped out.  -Post hoc sub-analysis performed.  -No differences in baseline characteristics for patients who completed f/u vs non completers. Nor, in preliminary outcome measures for supporters vs other. (Supplementary Tables). |
|  | Fortmann et al [62], 2017 |  | X |  | “A worst-case scenario sensitivity analysis is presented in Supplement A.” | -Attrition is reported for each outcome in Table 2. -However, Reasons for attrition are not provided.  Provided and reported transparently, also analysed ITT.  -Sensitivity analysis was done. |
|  | Moussa et al [58], 2013 |  | X |  | “One hundred percent (n = 46) of the participants completed the intervention group (n = 23) and comparison group (n = 23) sessions and no participants dropped from the program. Missing data were minimal, and no participants (n = 0) were excluded from analysis”.  “All participants completed each of the sessions, and missing data were minimal, therefore data for all 46 participants (intervention group (n=23) and comparison group (n=23)) were analysed according to the original randomization scheme” | -Dropout rates were zero.  -Missing data were minimal, and no participants (n = 0) were excluded from analysis. |
|  | Nelson et al [61], 2021 |  | X |  | “Measure completion rate was more than 90% at each of the follow-up assessments and did not differ across conditions (Fig. 1).”  “We employed multiple imputation with chained equations to address missing data (m 5 1,000 imputed data sets) and included all randomized participants in analyses.” | -Attrition rate was modest (max 10%).  -Reasons for attrition were not reported. proportion of participants who were excluded is low and it is unlikely to have a great influence on outcome data.  -Missing data have been imputed using appropriate methods. |
|  | Porter et al [55], 2009 |  | X |  | Main outcome: “face-validity and impact evaluation” | -No attrition is reported. And the outcome data for each main outcome appears to be complete. |
|  | Robertson et al [56], 2007 | X |  |  | “Analysis was completed for 29 of the 33 original intervention group participants. Of the 4 participants who were not included in the analysis, 1 never logged on to the Web site and the other 3 logged on but did not enter data.”  “Demographic data were not available for 3 intervention group members. No demographic data were available for the control participants.” | -Attrition rate was low (4/33).  -Details given about reasons for dropping out of study.  -However, due to incomplete demographic data, unable to accurately report group differences between completers and non-completers.  -Additionally, incomplete data for control group. |
|  | Ruggiero et al [57], 2014 | X |  |  | “A total of 69 participants were eligible for the intervention study (23/69 were lost to follow-up; 5 declined participation), and 41 consented and provided baseline assessments. There were four additional participants that withdrew prior to logging in at all; 37 participated in the intervention.”  “In order to take into consideration, the correlated nature of longitudinal data, and the fact that observations over time are nested within individuals, we performed a series of mixed-effects analyses with time as a single repeated measures factor. This approach is also quite compatible with an intent-to-treat approach to data analysis, as it does not require that respondents have complete data at all waves to be included in the analysis.” | -A high proportion of dropouts occurred, with reasons not stated.  -Additionally, differences in characteristics not provided between completers and non-completers. |
|  | Whittemore et al [60], 2020 |  | X |  | Attendance was high at 89% across all sessions and attrition was low at 6.4% (*n* = 3) at 6 mo. | -Attrition rate was low after intervention randomisation.  -Missing data have been imputed using appropriate methods. |
| Selective outcome reporting | Aligholipour et al [59], 2019 | X |  |  |  | -Registered trial protocol irct.21196  - However primary outcomes differ slightly in registered protocol (FBS, HbA1c, self-care) and paper (self-care mentioned as only primary though FBS reported) |
|  | Arora et al [53], 2012 |  | X |  |  | -The study’s pre-specified (primary and secondary) outcomes that are of interest have been reported in the pre-specified way. |
|  | Burner et al [54], 2017 |  |  | X |  | -All relevant outcomes in the methods section are reported in the results section.  -However, since the study protocol is not available, we don’t have sufficient evidence to make judgement of low risk or high risk. |
|  | Fortmann et al [62], 2017 |  | X |  |  | -Details of primary outcome match the registered protocol NCT01749176. |
|  | Moussa et al [58], 2013 |  |  | X |  | -All relevant outcomes in the methods section are reported in the results section.  -However, since the study protocol is not available, we don’t have sufficient evidence to make judgement of low risk or high risk. |
|  | Nelson et al [61], 2021 |  | X |  |  | -Outcomes match those recorded in clinical trial registration NCT02409329.  -Self-efficacy is added to paper. |
|  | Porter et al [55], 2009 |  | X |  | Main outcome: “face-validity and impact evaluation” | -The study’s pre-specified outcomes have been reported |
|  | Robertson et al [56], 2007 |  |  | X | “This was a pilot study with a convenience sample.”  “Demographic data were not available for 3 intervention group members. No demographic data were available for the control participants.” | -The study’s pre-specified (primary and secondary) outcomes that are of interest have been reported.  -Demographic data not available for control participants. |
|  | Ruggiero et al [57], 2014 |  | X |  | “The objective of the study was to examine the acceptability, usage, and preliminary outcome of a virtual world intervention, Diabetes Island, in low-income African Americans with type 2 diabetes.” | -The study’s pre-specified (primary and secondary) outcomes that are of interest have been reported in the pre-specified way. |
|  | Whittemore et al [60], 2020 | X |  |  |  | -A few differences to the protocol –  2 day diet history and accelerometer assessed PA in protocol but not reported in paper (different measures) |
| Other source of bias | Aligholipour et al [59], 2019 | X |  |  | “Before the education the study groups differed significantly from each other regarding patients’ self-care activities scores for the dimensions of diet and blood sugar testing.”  “In this study, the study sample was limited to patients with insulin-dependent diabetes referred to the emergency department and the clinic of Sina Hospital in Tabriz in July-September 2017. Therefore, it should be cautious in generalizing the results.” | -ANCOVA was used to control the pre-test effects on post-test scores.  -Participants in control group received paper-based educational materials.  -Recruitment procedure might limit generalizability.  -No strong source of other bias. |
|  | Arora et al [53], 2012 | X |  |  | “Subjects were eligible for inclusion if they were (1) ‡ 18 years old, (2) had diabetes, (3) had a text message–capable mobile phone, (4) knew how to receive text messages, and (5) spoke and read English or Spanish.” | -All participants needed to have a mobile phone (with text-message capabilities); this may limit generalizability.  -Small sample size (no formal sample size calculation performed due to proof-of-concept study), short follow-up which prevented evaluating a measurable change in HbA1c.  -Limited details describing health behaviour measures relating to diet, exercise and foot checks. |
|  | Burner et al [54], 2017 | X |  |  | “While in the ED, patients were screened for eligibility based on these criteria: having a text-capable mobile phone, being comfortable with sending and receiving texts, and having a glycosylated hemoglobin A1c (HbA1c) of ≥8, which falls into the ADA ‘‘Take Action’’ range.” | -The characteristics of such patients might have influenced the efficacy of the intervention and its generalizability.  -Small sample size.  -No strong source of other bias. |
|  | Fortmann et al [62], 2017 |  | X |  | All participants received a blood glucose meter (OneTouch Verio Meter; LifeScan, Inc., Milpitas, CA), testing strips, and instructions on use.  Second, it is unknown how much of the intervention content was received (i.e., read or comprehended) by participants. Nonetheless, the number of blood glucose values texted in may be construed as an indirect indicator of intervention engagement. | -No strong source of other bias. |
|  | Moussa et al [58], 2013 | X |  |  | “The surveys were developed by the author based on the information presented in the eCare We Care tutorials and were validated by two experts in diabetes education and eCare We Care.”  “The generalizability of study findings is limited as convenience sampling was used and the study was conducted in one geographical setting.” | -Instrument reliability and validity of diabetes knowledge survey not conducted.  -Validated performed by “two experts in diabetes education” without reference.  -Small convenience sample. |
|  | Nelson et al [61], 2021 | X |  |  | Participants were compensated up to $210 for completing all study measures. To encourage retention, we also sent birthday cards and provided study-branded magnets, water bottles, and t-shirts at follow-up assessments. The last follow-up assessment was completed April 2019. | -Monetary and gifts provision could promote incentives and expectation bias. |
|  | Porter et al [55], 2009 | X |  |  |  | -Small sample size n=22.  -No formal sample size calculation performed. |
|  | Robertson et al [56], 2007 | X |  |  | “This was a pilot study with a convenience sample.”  “Demographic data were not available for 3 intervention group members. No demographic data were available for the control participants.”  “Participants self-reported their dietary intake”. | -Small convenience sample. Insufficient details about local population!  -Demographic data not available for 3 intervention group participants and all control participants. Therefore, unable to compare group characteristics and adjust accordingly / where necessary. |
|  | Ruggiero et al [57], 2014 | X |  |  | “The participants received US $25 for completion of each assessment at the research site”.  “Small sample size, quasi-experimental design, and sample selection”. “Lack of a comparison group necessitates cautious interpretation of the findings pending further research”. | -Monetary provision could promote incentives and expectation bias. |
|  | Whittemore et al [60], 2020 | X |  |  | All participants received a gift card for a department store after data collection; each participant received $200Mexican pesos (∼$10 USD) at baseline, $300 Mexican pesos (∼$15 USD) at 3 mo, and $400 Mexican pesos (∼$20 USD) at 6 mo. | -Monetary provision could promote incentives and expectation bias. |
